# Transient Pressure Behavior of CBM Wells during the Injection Fall-Off Test Considering the Quadratic Pressure Gradient

**DOI:** 10.3390/nano14131070

**Published:** 2024-06-22

**Authors:** Wei Gu, Jiaqi Wu, Zheng Sun

**Affiliations:** 1 State Key Laboratory for Fine Exploration and Intelligent Development of Coal Resources, China University of Mining and Technology, Xuzhou 221116, China; guwei@cumt.edu.cn; 2 CNOOC Research Institute Co., Ltd., Beijing 100028, China; wujq13@cnooc.com.cn

**Keywords:** natural nanomaterial, injection fall-off test, quadratic gradient term, nonlinear model, transient pressure behavior

## Abstract

Conventional coalbed methane (CBM) reservoir models for injection fall-off testing often disregard the quadratic pressure gradient’s impact. This omission leads to discrepancies in simulating the transient behavior of formation fluids and extracting critical reservoir properties. Accurate determination of permeability, storability, and other properties is crucial for effective reservoir characterization and production forecasting. Inaccurate estimations can lead to suboptimal well placement, ineffective production strategies, and ultimately, missed economic opportunities. To address this shortcoming, we present a novel analytical model that explicitly incorporates the complexities of the quadratic pressure gradient and dual-permeability flow mechanisms, prevalent in many CBM formations where nanopores are rich, presenting a kind of natural nanomaterial. This model offers significant advantages over traditional approaches. By leveraging variable substitution, it facilitates the derivation of analytical solutions in the Laplace domain, subsequently converted to real-space solutions for practical application. These solutions empower reservoir engineers to generate novel type curves, a valuable tool for analyzing wellbore pressure responses during injection fall-off tests. By identifying distinct flow regimes within the reservoir based on these type curves, engineers gain valuable insights into the dynamic behavior of formation fluids. This model goes beyond traditional approaches by investigating the influence of the quadratic pressure gradient coefficient, inter-porosity flow coefficient, and storability ratio on the pressure response. A quantitative comparison with traditional models further elucidates the key discrepancies caused by neglecting the quadratic pressure gradient. The results demonstrate the proposed model’s ability to accurately depict the non-linear flow behavior observed in CBM wells. This translates to more reliable pressure and pressure derivative curves that account for the impact of the quadratic pressure gradient.

## 1. Introduction

Fueled by the relentless growth of the global economy, the demand for energy continues to outpace the production capabilities of conventional natural gas resources. Coalbed methane (CBM) reservoirs, with their vast potential for gas storage and widespread geographic distribution, have emerged as a promising alternative to address this growing energy gap. CBM development has achieved notable success in recent years, playing an increasingly crucial role in supplementing and ultimately supplanting conventional oil and gas production in some regions, such as the eastern edge of the Ordos basin, Western Guizhou, China [1,2]. As a result, efficient development of CBM resources is considered a key strategic approach to alleviate the global pressure on energy supplies. Coal seams are fundamentally distinct from conventional reservoirs in terms of their composition, internal structure, nanoscale pores, and the interplay between their physical and mechanical properties [3]. Coal beds have abundant nanopores, with a pore size range of 10~100 nm [4,5,6], and can be regarded as a kind of natural nanomaterial, further aggravating the complexity in accurately describing the fluid flow behavior. This distinction is further emphasized by the occurrence and production mechanisms of CBM, which differ significantly from those governing the behavior of natural gas in sandstone reservoirs [7,8,9]. Due to their inherent dual-porosity nature, consisting of a matrix system with a large network of cleats (natural fractures) developed and retained within the coal seam, CBM reservoirs present a more complex flow environment compared to their conventional counterparts [10]. Thorough understanding and characterization of these critical CBM reservoir parameters are essential prerequisites for the scientific and rational development of CBM fields. Among these parameters, coal reservoir permeability reigns supreme in importance. It serves as the cornerstone for formulating development plans, wellbore pattern deployment strategies, hydraulic fracturing design optimization, and the allocation of production quotas across the reservoir [11,12]. Consequently, accurate determination of coal reservoir permeability is paramount for successful CBM development.

Well testing remains a crucial method for acquiring critical coal reservoir characteristic parameters, such as permeability, storability, and formation pressure. However, the unique characteristics of coal reservoirs, including low permeability, low formation pressure, susceptibility to damage due to soft rock, and pronounced heterogeneity, pose significant challenges to well test design and interpretation. The primary storage mechanism for CBM is adsorption within the coal matrix itself, a process governed by complex physical interactions between the gas molecules and the coal micropore network [13,14]. Utilizing conventional well testing methods during the initial opening period, known as the “bullhead” test, can readily induce unwanted co-production of water and gas. Furthermore, the inherently low reservoir permeability leads to extended pressure recovery times after a well shut-in, making it difficult to accurately determine the true formation pressure during testing. Consequently, traditional well test analysis methods often struggle to provide accurate interpretations of coal reservoir characteristic parameters. The injection fall-off testing method is the most widely employed approach for CBM well testing [15,16]. Extensive research has been conducted on CBM flow theory, encompassing analytical, semi-analytical, and numerical simulation models. These models have been applied to investigate nonlinear flow problems associated with vertical wells, fractured vertical wells, horizontal wells, and multi-stage fractured horizontal wells, providing valuable insights into wellbore pressure behavior under various completion scenarios [11,12,13,14,15,16,17]. While numerical simulation methods offer valuable insights, their pre-processing stage can be intricate, requiring significant time and expertise to prepare the model for simulation. Additionally, computational times can be significant, especially for complex reservoir models. Compared to analytical methods, numerical simulation approaches often present a higher level of operational complexity for well test analysis, requiring specialized software and a deep understanding of reservoir engineering principles. Additionally, numerical models may not accurately capture the early-time wellbore storage and skin effects that can influence pressure behavior, particularly during the initial flow period following a shut-in. Analytical and semi-analytical models, while offering advantages in terms of computational efficiency, often rely on simplifying assumptions to facilitate solutions [3,14,18]. In comparison with analytical models, numerical models can readily incorporate newly emerging mechanisms [19,20,21,22]. A common simplification involves neglecting the quadratic pressure gradient term. This omission linearizes the model, making it easier to solve mathematically, but it also introduces discrepancies between the simulated flow behavior and actual reservoir conditions. In recent years, numerous researchers have emphasized the inherent quadratic pressure dependence of formation fluid flow, highlighting the importance of incorporating this term in well testing models to improve the accuracy of pressure transient analysis [23,24,25,26]. Nonlinear flow models that account for the quadratic pressure gradient have been shown to provide more accurate simulations of formation fluid flow behavior, capturing the influence of pressure-dependent permeability and other non-linear effects [27,28,29,30]. Despite the growing body of research on nonlinear flow theory with quadratic pressure gradients, the overall theoretical framework remains less comprehensive compared to its counterpart in conventional linear flow theory. Existing models for nonlinear flow with a quadratic pressure gradient primarily focus on homogeneous reservoirs, assuming uniform properties throughout the formation [31,32,33,34]. These models are generally inadequate for analyzing the transient pressure response observed during CBM well injection fall-off tests, as they fail to account for the inherent heterogeneity present in coal formations.

This work proposes a novel analytical model to simulate the transient pressure response of CBM wells during injection fall-off tests. This model incorporates two key features often neglected in conventional approaches: the quadratic pressure gradient and dual-permeability flow behavior, both of which are prevalent in CBM formations [35,36]. To address the non-linear flow issue arising from the quadratic pressure gradient term, we present an efficient solution methodology. By employing variable substitution, Laplace transformation, and Stefest numerical inversion, we transform the non-linear mathematical model into a linear form, enabling the derivation of an analytical solution. Utilizing this solution, we generate novel type curves that visualize distinct flow regimes within the reservoir during the test. We further investigate the influence of critical parameters on the transient pressure response, providing valuable insights into reservoir behavior. Finally, a quantitative comparison with traditional models highlights the key discrepancies caused by neglecting the quadratic pressure gradient. This comparison underscores the improved accuracy and robustness of the proposed model.

## 2. Methodology

### 2.1. Physical Model Assumption

**Figure 1:**
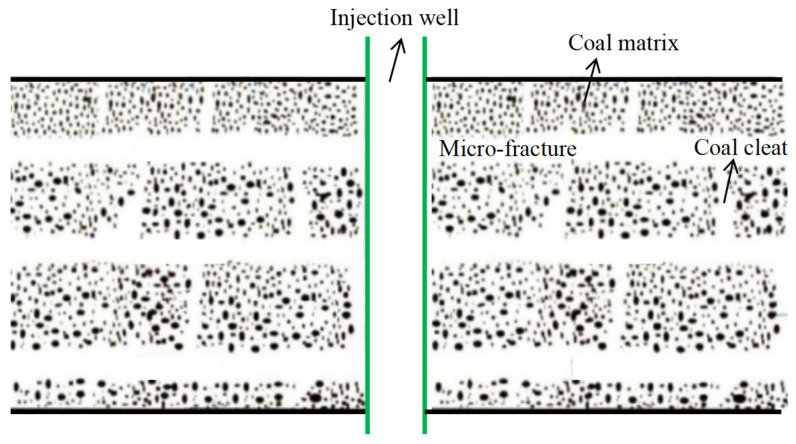
Schematic of a CBM reservoir.

**Figure 2:**
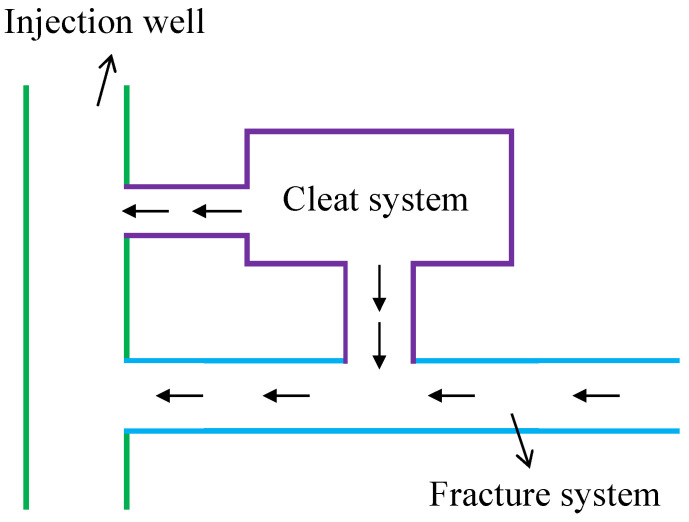
Flow scheme of a vertical well in a CBM reservoir.

The coal seam under investigation can be conceptualized as a complex geological structure, often referred to as a “dual-porosity, dual-permeability” system. This system comprises three distinct components: a matrix system, a cleat system, and a natural fracture system, as illustrated in Figure 1. Each of these components contributes to the overall storage and flow capacity of the reservoir. To understand the flow behavior within this intricate reservoir, an injection fall-off test is conducted in a vertical well, as depicted in Figure 2. The following key assumptions underpin the development of the mathematical model:

(1) Slightly compressible fluid and coal matrix: Both the formation fluid and the coal matrix exhibit slight compressibility, characterized by a constant compressibility coefficient. This implies that the volume of both the fluid and the rock can be slightly compressed or expanded in response to changes in pressure.
(2) Cleat and fracture properties: The permeability within the cleat and natural fracture systems is assumed to be constant and isotropic in the horizontal plane. Isotropy in this context signifies that the permeability does not vary with direction within the horizontal plane.
(3) Isothermal Darcy flow: Fluid flow within the cleat and fracture network is governed by the isothermal Darcy’s Law, neglecting the influence of gravity. This assumption implies that the flow is driven by pressure differences within the reservoir and that temperature variations are not considered significant for the purposes of this model.
(4) Wellbore storage and skin effects: The model incorporates the effects of wellbore storage and skin effect, which can significantly impact pressure behavior during well testing. Wellbore storage refers to the volume of fluid contained within the wellbore itself, while skin effect represents the additional pressure drop or gain that occurs near the wellbore due to formation damage or stimulation.
(5) Non-linear flow with quadratic pressure gradient: The model accounts for the non-linear flow behavior arising from the quadratic pressure gradient, a critical factor in CBM reservoirs. The quadratic pressure gradient refers to the phenomenon where the rate of change of pressure with respect to distance is not constant but rather increases with increasing pressure. This non-linearity can have a significant impact on the pressure response observed during well testing.

### 2.2. Mathematical Model

Pioneering work by Hnjord and Aadnoy in 1989 established a foundational concept for well testing analysis [19]. They derived a differential control equation governing liquid flow within homogeneous underground reservoirs. This equation incorporated a critical term representing the product of the squared pressure gradient and the liquid compressibility coefficient. This term, known as the quadratic pressure gradient term, introduces a significant non-linearity into the governing partial differential equation. In essence, the presence of the quadratic pressure gradient term underscores the inherent non-linear nature of fluid flow within the reservoir itself. This non-linearity arises from the interplay between pressure changes and the compressibility of the formation fluids.

(1) $$\left(\frac{\partial^{2}p}{\partial x^{2}}+\frac{\partial^{2}p}{\partial y^{2}}+\frac{k_{z}}{k_{h}}\frac{\partial^{2}p}{\partial z^{2}}\right)+C_{\rho }\left[{\left(\frac{\partial p}{\partial x}\right)}^{2}+{\left(\frac{\partial p}{\partial y}\right)}^{2}+\frac{k_{z}}{k_{h}}{\left(\frac{\partial p}{\partial z}\right)}^{2}\right]=\frac{\mu \phi C_{t}}{k_{h}}\frac{\partial p}{\partial t}\;\left(\frac{\partial^{2}p}{\partial x^{2}}+\frac{\partial^{2}p}{\partial y^{2}}+\frac{k_{z}}{k_{h}}\frac{\partial^{2}p}{\partial z^{2}}\right)+C_{\rho }\left[{\left(\frac{\partial p}{\partial x}\right)}^{2}+{\left(\frac{\partial p}{\partial y}\right)}^{2}+\frac{k_{z}}{k_{h}}{\left(\frac{\partial p}{\partial z}\right)}^{2}\right]=\frac{\mu \phi C_{t}}{k_{h}}\frac{\partial p}{\partial t}$$

#### 2.2.1. Nonlinear Governing Equation

Leveraging the previously established physical model assumptions, we proceed to develop a comprehensive mathematical model for simulating the transient seepage behavior of a single CBM well. This model is formulated within the framework of a cylindrical radial coordinate system, which is a well-suited geometrical representation for vertical wells in axisymmetric reservoirs. The model explicitly incorporates the critical effects of the quadratic pressure gradient, wellbore storage, and skin effect. The quadratic pressure gradient term accounts for the non-linear flow behavior observed in CBM formations due to the interplay between pressure changes and the compressibility of formation fluids. Wellbore storage refers to the volume of fluid contained within the wellbore itself, which can influence the pressure response during well testing. The skin effect represents the additional pressure drop or gain that occurs near the wellbore due to formation damage or stimulation, impacting the flow behavior in the near-wellbore region. By incorporating these key factors, the proposed model aims to provide a more accurate and realistic representation of pressure behavior during injection fall-off tests in CBM wells.

The partial differential control equation for seepage in the natural fracture system is as follows:

(2) $$\frac{\partial^{2}p_{F}}{\partial r^{2}}+\frac{1}{r}\frac{\partial p_{F}}{\partial r}+C_{\rho }{\left(\frac{\partial p_{F}}{\partial r}\right)}^{2}+\alpha \frac{k_{f}}{k_{F}}\left(p_{f}-p_{F}\right)=\frac{\phi_{F}\mu_{w}c_{\mathrm{tF}}}{3.6k_{F}}\frac{\partial p_{F}}{\partial t}\;\frac{\partial^{2}p_{F}}{\partial r^{2}}+\frac{1}{r}\frac{\partial p_{F}}{\partial r}+C_{\rho }{\left(\frac{\partial p_{F}}{\partial r}\right)}^{2}+\alpha \frac{k_{f}}{k_{F}}\left(p_{f}-p_{F}\right)=\frac{\phi_{F}\mu_{w}c_{\mathrm{tF}}}{3.6k_{F}}\frac{\partial p_{F}}{\partial t}$$

The partial differential control equation for seepage in the cleat system is as follows:

(3) $$\frac{\partial^{2}p_{f}}{\partial r^{2}}+\frac{1}{r}\frac{\partial p_{f}}{\partial r}+C_{\rho }{\left(\frac{\partial p_{f}}{\partial r}\right)}^{2}-\alpha \frac{k_{f}}{k_{F}}\left(p_{f}-p_{F}\right)=\frac{\phi_{f}\mu_{w}c_{\mathrm{tf}}}{3.6k_{f}}\frac{\partial p_{f}}{\partial t}\;\frac{\partial^{2}p_{f}}{\partial r^{2}}+\frac{1}{r}\frac{\partial p_{f}}{\partial r}+C_{\rho }{\left(\frac{\partial p_{f}}{\partial r}\right)}^{2}-\alpha \frac{k_{f}}{k_{F}}\left(p_{f}-p_{F}\right)=\frac{\phi_{f}\mu_{w}c_{\mathrm{tf}}}{3.6k_{f}}\frac{\partial p_{f}}{\partial t}$$

The initial condition is as follows:

(4) $$p_{F}{|}_{t=0}=p_{f}{|}_{t=0}=p_{i}\;p_{F}{|}_{t=0}=p_{f}{|}_{t=0}=p_{i}$$

The inner boundary conditions are as follows:

(5) $$\frac{h}{\mu_{w}}r\left(k_{F}\frac{\partial p_{F}}{\partial r}+k_{f}\frac{\partial p_{f}}{\partial r}\right){|}_{r=r_{w}}=1.842\times 10^{-3}qB_{w}+0.04421C_{s}\frac{dp_{w}}{dt}\;\frac{h}{\mu_{w}}r\left(k_{F}\frac{\partial p_{F}}{\partial r}+k_{f}\frac{\partial p_{f}}{\partial r}\right){|}_{r=r_{w}}=1.842\times 10^{-3}qB_{w}+0.04421C_{s}\frac{dp_{w}}{dt}$$

(6) $$p_{w}=\left[p_{F}-Sr\left(\frac{\partial p_{F}}{\partial r}+\frac{\partial p_{f}}{\partial r}\right)\right]{|}_{r=r_{w}}\;p_{w}=\left[p_{F}-Sr\left(\frac{\partial p_{F}}{\partial r}+\frac{\partial p_{f}}{\partial r}\right)\right]{|}_{r=r_{w}}$$

The outer boundary conditions are as follows:

Infinite outer boundary

(7) $$\underset{r\to \infty }{\lim}p_{F}=\underset{r\to \infty }{\lim}p_{f}=p_{i}\;\underset{r\to \infty }{\lim}p_{F}=\underset{r\to \infty }{\lim}p_{f}=p_{i}$$

Closed boundary

(8) $$\frac{\partial p_{F}}{\partial r}{|}_{r=r_{e}}=\frac{\partial p_{f}}{\partial r}{|}_{r=r_{e}}\;\frac{\partial p_{F}}{\partial r}{|}_{r=r_{e}}=\frac{\partial p_{f}}{\partial r}{|}_{r=r_{e}}$$

Constant pressure boundary

(9) $$p_{F}{|}_{r=r_{e}}=p_{f}{|}_{r=r_{e}}=p_{i}\;p_{F}{|}_{r=r_{e}}=p_{f}{|}_{r=r_{e}}=p_{i}$$

where, *p*_F_ is the pressure in the natural fracture system, MPa; *p*_f_ is the pressure in the cleat system, MPa; *p*_i_ is the initial pressure of the formation, MPa; *p*_w_ is the bottom-hole pressure, MPa; *t* is the time, h; *μ*_w_ is the water viscosity, mPa⋅s; *B*_w_ is the water volume coefficient, m^3^/m^3^; *q* is surface production rate, m^3^/d; *r* is the radial distance, m; *r*_e_ is the distance of the external boundary, m; *k*_F_ is the permeability of natural fractures, mD; *k*_f_ is the permeability of cleats, mD; *ϕ*_F_ is the porosity of natural fractures; *ϕ*_f_ is the porosity of cleats; *α* is the geometric shape factor of the matrix block, m^−2^; *S* is the skin factor; *C*_s_ is the wellbore storage factor, m^3^/MPa; *C*_tF_ is the total compressibility coefficient of the natural fracture system, MPa^−1^; *C*_tf_ is the total compressibility coefficient of the cleat system, MPa^−1^; *C*_ρ_ is the isothermal compressibility coefficient of the water phase, MPa^−1^.

#### 2.2.2. Dimensionless Mathematical Model

To make the equations homogeneous, the definitions and tabulations of dimensionless variables are presented in Table 1.

By substituting the above dimensionless parameters and definitions into Equations (2)–(8), the following dimensionless mathematical model can be obtained.

The partial differential control equation for seepage in the natural fracture system is as follows:

(10) $$\kappa \left[\frac{\partial^{2}p_{\mathrm{FD}}}{\partial {r_{D}}^{2}}+\frac{1}{r_{D}}\frac{\partial p_{\mathrm{FD}}}{\partial r_{D}}-\beta {\left(\frac{\partial p_{\mathrm{FD}}}{\partial r_{D}}\right)}^{2}\right]-\lambda e^{-2S}\left(p_{\mathrm{FD}}-p_{\mathrm{fD}}\right)=\frac{\omega_{F}}{C_{D}e^{2S}}\frac{\partial p_{\mathrm{FD}}}{\partial \left(t_{D}/C_{D}\right)}\;\kappa \left[\frac{\partial^{2}p_{\mathrm{FD}}}{\partial {r_{D}}^{2}}+\frac{1}{r_{D}}\frac{\partial p_{\mathrm{FD}}}{\partial r_{D}}-\beta {\left(\frac{\partial p_{\mathrm{FD}}}{\partial r_{D}}\right)}^{2}\right]-\lambda e^{-2S}\left(p_{\mathrm{FD}}-p_{\mathrm{fD}}\right)=\frac{\omega_{F}}{C_{D}e^{2S}}\frac{\partial p_{\mathrm{FD}}}{\partial \left(t_{D}/C_{D}\right)}$$

The partial differential control equation for seepage in the cleat system is as follows:

(11) $$\left(1-\kappa \right)\left[\frac{\partial^{2}p_{\mathrm{fD}}}{\partial {r_{D}}^{2}}+\frac{1}{r_{D}}\frac{\partial p_{\mathrm{fD}}}{\partial r_{D}}-\beta {\left(\frac{\partial p_{\mathrm{fD}}}{\partial r_{D}}\right)}^{2}\right]+\lambda e^{-2S}\left(p_{\mathrm{FD}}-p_{\mathrm{fD}}\right)=\frac{\omega_{f}}{C_{D}e^{2S}}\frac{\partial p_{\mathrm{fD}}}{\partial \left(t_{D}/C_{D}\right)}\;\left(1-\kappa \right)\left[\frac{\partial^{2}p_{\mathrm{fD}}}{\partial {r_{D}}^{2}}+\frac{1}{r_{D}}\frac{\partial p_{\mathrm{fD}}}{\partial r_{D}}-\beta {\left(\frac{\partial p_{\mathrm{fD}}}{\partial r_{D}}\right)}^{2}\right]+\lambda e^{-2S}\left(p_{\mathrm{FD}}-p_{\mathrm{fD}}\right)=\frac{\omega_{f}}{C_{D}e^{2S}}\frac{\partial p_{\mathrm{fD}}}{\partial \left(t_{D}/C_{D}\right)}$$

Initial condition

(12) $$p_{\mathrm{FD}}{|}_{\left(t_{D}/C_{D}\right)=0}=p_{\mathrm{fD}}{|}_{\left(t_{D}/C_{D}\right)=0}=0\;p_{\mathrm{FD}}{|}_{\left(t_{D}/C_{D}\right)=0}=p_{\mathrm{fD}}{|}_{\left(t_{D}/C_{D}\right)=0}=0$$

Inner boundary condition

(13) $$\frac{\partial p_{\mathrm{FD}}}{\partial \left(t_{D}/C_{D}\right)}{|}_{r_{D}=1}-\left(r_{D}\kappa \frac{\partial p_{\mathrm{FD}}}{\partial r_{D}}+r_{D}\left(1-\kappa \right)\frac{\partial p_{\mathrm{fD}}}{\partial r_{D}}\right){|}_{r_{D}=1}=1\;\;\frac{\partial p_{\mathrm{FD}}}{\partial \left(t_{D}/C_{D}\right)}{|}_{r_{D}=1}-\left(r_{D}\kappa \frac{\partial p_{\mathrm{FD}}}{\partial r_{D}}+r_{D}\left(1-\kappa \right)\frac{\partial p_{\mathrm{fD}}}{\partial r_{D}}\right){|}_{r_{D}=1}=1$$

The outer boundary conditions are as follows:

In the case of infinite,

(14) $$\underset{r_{D}\to \infty }{\lim}p_{\mathrm{FD}}\left(r_{D},t_{D}/C_{D}\right)=\underset{r_{D}\to \infty }{\lim}p_{\mathrm{fD}}\left(r_{D},t_{D}/C_{D}\right)=0\;\underset{r_{D}\to \infty }{\lim}p_{\mathrm{FD}}\left(r_{D},t_{D}/C_{D}\right)=\underset{r_{D}\to \infty }{\lim}p_{\mathrm{fD}}\left(r_{D},t_{D}/C_{D}\right)=0$$

In the case of closed,

(15) $$\frac{\partial p_{\mathrm{FD}}}{\partial r_{D}}{|}_{r_{D}=r_{\mathrm{eD}}}=\frac{\partial p_{\mathrm{fD}}}{\partial r_{D}}{|}_{r_{D}=r_{\mathrm{eD}}}\;\frac{\partial p_{\mathrm{FD}}}{\partial r_{D}}{|}_{r_{D}=r_{\mathrm{eD}}}=\frac{\partial p_{\mathrm{fD}}}{\partial r_{D}}{|}_{r_{D}=r_{\mathrm{eD}}}$$

In the case of constant pressure,

(16) $$p_{\mathrm{FD}}{|}_{r_{D}=r_{\mathrm{eD}}}=p_{\mathrm{fD}}{|}_{r_{D}=r_{\mathrm{eD}}}=0\;p_{\mathrm{FD}}{|}_{r_{D}=r_{\mathrm{eD}}}=p_{\mathrm{fD}}{|}_{r_{D}=r_{\mathrm{eD}}}=0$$

#### 2.2.3. Solution to Mathematical Model

The inclusion of the quadratic pressure gradient term in Equations (9) and (10) renders them non-linear partial differential equations. Solving such equations directly can be mathematically intricate. To circumvent this challenge and achieve a tractable analytical solution, we leverage the established technique of variable substitution employed in previous studies [22]. This technique involves introducing a transformation of the dimensionless pressure variable. The specific transformation will be presented in the following section.

(17) $$p_{\mathrm{FD}}=-\frac{1}{\beta }\ln\left(\zeta_{F}+1\right)\;p_{\mathrm{FD}}=-\frac{1}{\beta }\ln\left(\zeta_{F}+1\right)$$

Using Equation (16) to transform the set of dimensionless equations, Equations (9)–(15), the partial differential control equation for seepage in the natural fracture system is as follows:

(18) $$\kappa \left(\frac{\partial^{2}\zeta_{F}}{\partial {r_{D}}^{2}}+\frac{1}{r_{D}}\frac{\partial \zeta_{F}}{\partial r_{D}}\right)-\lambda e^{-2S}\left[-\frac{1}{\beta }\ln\left(\zeta_{F}+1\right)+\frac{1}{\beta }\ln\left(\zeta_{f}+1\right)\right]=\frac{\omega_{F}}{C_{D}e^{2S}}\frac{\partial \zeta_{F}}{\partial T_{D}}\;\kappa \left(\frac{\partial^{2}\zeta_{F}}{\partial {r_{D}}^{2}}+\frac{1}{r_{D}}\frac{\partial \zeta_{F}}{\partial r_{D}}\right)-\lambda e^{-2S}\left[-\frac{1}{\beta }\ln\left(\zeta_{F}+1\right)+\frac{1}{\beta }\ln\left(\zeta_{f}+1\right)\right]=\frac{\omega_{F}}{C_{D}e^{2S}}\frac{\partial \zeta_{F}}{\partial T_{D}}$$

The partial differential control equation for seepage in the cleat system is as follows:

(19) $$\left(1-\kappa \right)\left(\frac{\partial^{2}\zeta_{f}}{\partial {r_{D}}^{2}}+\frac{1}{r_{D}}\frac{\partial \zeta_{f}}{\partial r_{D}}\right)+\lambda e^{-2S}\left[-\frac{1}{\beta }\ln\left(\zeta_{F}+1\right)+\frac{1}{\beta }\ln\left(\zeta_{f}+1\right)\right]=\frac{\omega_{f}}{C_{D}e^{2S}}\frac{\partial \zeta_{f}}{\partial T_{D}}\;\left(1-\kappa \right)\left(\frac{\partial^{2}\zeta_{f}}{\partial {r_{D}}^{2}}+\frac{1}{r_{D}}\frac{\partial \zeta_{f}}{\partial r_{D}}\right)+\lambda e^{-2S}\left[-\frac{1}{\beta }\ln\left(\zeta_{F}+1\right)+\frac{1}{\beta }\ln\left(\zeta_{f}+1\right)\right]=\frac{\omega_{f}}{C_{D}e^{2S}}\frac{\partial \zeta_{f}}{\partial T_{D}}$$

Initial condition

(20) $$\zeta_{F}{|}_{T_{D}=0}=\zeta_{f}{|}_{T_{D}=0}=0\;\zeta_{F}{|}_{T_{D}=0}=\zeta_{f}{|}_{T_{D}=0}=0$$

Inner boundary condition

(21) $$-\frac{1}{\beta \left(\zeta_{F}+1\right)}\frac{\partial \zeta_{F}}{\partial T_{D}}{|}_{r_{D}=1}+\left[\kappa \frac{1}{\beta \left(\zeta_{F}+1\right)}\frac{\partial \zeta_{F}}{\partial r_{D}}+\left(1-\kappa \right)\frac{1}{\beta \left(\zeta_{f}+1\right)}\frac{\partial \zeta_{f}}{\partial r_{D}}\right]{|}_{r_{D}=1}=1\;-\frac{1}{\beta \left(\zeta_{F}+1\right)}\frac{\partial \zeta_{F}}{\partial T_{D}}{|}_{r_{D}=1}+\left[\kappa \frac{1}{\beta \left(\zeta_{F}+1\right)}\frac{\partial \zeta_{F}}{\partial r_{D}}+\left(1-\kappa \right)\frac{1}{\beta \left(\zeta_{f}+1\right)}\frac{\partial \zeta_{f}}{\partial r_{D}}\right]{|}_{r_{D}=1}=1$$

Outer boundary conditions

(22) $$\underset{r_{D}\to \infty }{\lim}\zeta_{F}=\underset{r_{D}\to \infty }{\lim}\zeta_{f}=0\;\underset{r_{D}\to \infty }{\lim}\zeta_{F}=\underset{r_{D}\to \infty }{\lim}\zeta_{f}=0$$

(23) $$\frac{\partial \zeta_{F}}{\partial r_{D}}{|}_{r_{D}=r_{\mathrm{eD}}}=\frac{\partial \zeta_{f}}{\partial r_{D}}{|}_{r_{D}=r_{\mathrm{eD}}}\;\frac{\partial \zeta_{F}}{\partial r_{D}}{|}_{r_{D}=r_{\mathrm{eD}}}=\frac{\partial \zeta_{f}}{\partial r_{D}}{|}_{r_{D}=r_{\mathrm{eD}}}$$

(24) $$\zeta_{F}{|}_{r_{D}=r_{\mathrm{eD}}}=\zeta_{f}{|}_{r_{D}=r_{\mathrm{eD}}}=0\;\zeta_{F}{|}_{r_{D}=r_{\mathrm{eD}}}=\zeta_{f}{|}_{r_{D}=r_{\mathrm{eD}}}=0$$

By introducing the Laplace transform based on *t*_D_/*C*_D_, we obtain:

(25) $$L[p_{\mathrm{FD}}(r_{D},t_{D}/C_{D})]={\bar{p}}_{\mathrm{FD}}\left(r_{D},u\right)=\int_{0}^{+\infty }p_{\mathrm{FD}}(r_{D},t_{D}/C_{D})e^{-ut_{D}/C_{D}}dt_{D}/C_{D}\;L[p_{\mathrm{FD}}(r_{D},t_{D}/C_{D})]={\bar{p}}_{\mathrm{FD}}\left(r_{D},u\right)=\int_{0}^{+\infty }p_{\mathrm{FD}}(r_{D},t_{D}/C_{D})e^{-ut_{D}/C_{D}}dt_{D}/C_{D}$$

(26) $$L[p_{\mathrm{fD}}(r_{D},t_{D}/C_{D})]={\bar{p}}_{\mathrm{fD}}\left(r_{D},u\right)=\int_{0}^{+\infty }p_{\mathrm{fD}}(r_{D},t_{D}/C_{D})e^{-ut_{D}/C_{D}}dt_{D}/C_{D}\;L[p_{\mathrm{fD}}(r_{D},t_{D}/C_{D})]={\bar{p}}_{\mathrm{fD}}\left(r_{D},u\right)=\int_{0}^{+\infty }p_{\mathrm{fD}}(r_{D},t_{D}/C_{D})e^{-ut_{D}/C_{D}}dt_{D}/C_{D}$$

By applying the Laplace transform to Equations (17)–(23), the set of equations becomes as follows:

The partial differential control equation for seepage in the natural fracture system is as follows:

(27) $$\frac{\partial^{2}{\bar{\zeta }}_{F}}{\partial {r_{D}}^{2}}+\frac{1}{r_{D}}\frac{\partial {\bar{\zeta }}_{F}}{\partial r_{D}}-\frac{\lambda e^{-2S}}{\kappa }\left(-\frac{1}{\beta }\frac{1}{{\bar{\zeta }}_{F}+1}+\frac{1}{\beta }\frac{1}{{\bar{\zeta }}_{f}+1}\right)=\frac{\omega_{F}}{\kappa C_{D}e^{2S}}u{\bar{\zeta }}_{F}\;\frac{\partial^{2}{\bar{\zeta }}_{F}}{\partial {r_{D}}^{2}}+\frac{1}{r_{D}}\frac{\partial {\bar{\zeta }}_{F}}{\partial r_{D}}-\frac{\lambda e^{-2S}}{\kappa }\left(-\frac{1}{\beta }\frac{1}{{\bar{\zeta }}_{F}+1}+\frac{1}{\beta }\frac{1}{{\bar{\zeta }}_{f}+1}\right)=\frac{\omega_{F}}{\kappa C_{D}e^{2S}}u{\bar{\zeta }}_{F}$$

The partial differential control equation for seepage in the cleat system is as follows:

(28) $$\frac{\partial^{2}{\bar{\zeta }}_{f}}{\partial {r_{D}}^{2}}+\frac{1}{r_{D}}\frac{\partial {\bar{\zeta }}_{f}}{\partial r_{D}}+\frac{\lambda e^{-2S}}{1-\kappa }\left(-\frac{1}{\beta }\frac{1}{{\bar{\zeta }}_{F}+1}+\frac{1}{\beta }\frac{1}{{\bar{\zeta }}_{f}+1}\right)=\frac{\omega_{f}}{\left(1-\kappa \right)C_{D}e^{2S}}u{\bar{\zeta }}_{f}\;\;\frac{\partial^{2}{\bar{\zeta }}_{f}}{\partial {r_{D}}^{2}}+\frac{1}{r_{D}}\frac{\partial {\bar{\zeta }}_{f}}{\partial r_{D}}+\frac{\lambda e^{-2S}}{1-\kappa }\left(-\frac{1}{\beta }\frac{1}{{\bar{\zeta }}_{F}+1}+\frac{1}{\beta }\frac{1}{{\bar{\zeta }}_{f}+1}\right)=\frac{\omega_{f}}{\left(1-\kappa \right)C_{D}e^{2S}}u{\bar{\zeta }}_{f}$$

Inner boundary condition

(29) $$-\frac{1}{\beta \left({\bar{\zeta }}_{F}+1\right)}u{\bar{\zeta }}_{F}{|}_{r_{D}=1}+\left[\frac{\kappa }{\beta \left({\bar{\zeta }}_{F}+1\right)}\frac{\partial {\bar{\zeta }}_{F}}{\partial r_{D}}+\frac{1-\kappa }{\beta \left({\bar{\zeta }}_{f}+1\right)}\frac{\partial {\bar{\zeta }}_{f}}{\partial r_{D}}\right]{|}_{r_{D}=1}=\frac{1}{u}\;-\frac{1}{\beta \left({\bar{\zeta }}_{F}+1\right)}u{\bar{\zeta }}_{F}{|}_{r_{D}=1}+\left[\frac{\kappa }{\beta \left({\bar{\zeta }}_{F}+1\right)}\frac{\partial {\bar{\zeta }}_{F}}{\partial r_{D}}+\frac{1-\kappa }{\beta \left({\bar{\zeta }}_{f}+1\right)}\frac{\partial {\bar{\zeta }}_{f}}{\partial r_{D}}\right]{|}_{r_{D}=1}=\frac{1}{u}$$

Outer boundary conditions

(30) $$\underset{r_{D}\to \infty }{\lim}{\bar{\zeta }}_{F}=\underset{r_{D}\to \infty }{\lim}{\bar{\zeta }}_{f}=0\;\underset{r_{D}\to \infty }{\lim}{\bar{\zeta }}_{F}=\underset{r_{D}\to \infty }{\lim}{\bar{\zeta }}_{f}=0$$

(31) $$\frac{\partial {\bar{\zeta }}_{F}}{\partial r_{D}}{|}_{r_{D}=r_{\mathrm{eD}}}=\frac{\partial {\bar{\zeta }}_{f}}{\partial r_{D}}{|}_{r_{D}=r_{\mathrm{eD}}}\;\frac{\partial {\bar{\zeta }}_{F}}{\partial r_{D}}{|}_{r_{D}=r_{\mathrm{eD}}}=\frac{\partial {\bar{\zeta }}_{f}}{\partial r_{D}}{|}_{r_{D}=r_{\mathrm{eD}}}$$

(32) $${\bar{\zeta }}_{F}{|}_{r_{D}=r_{\mathrm{eD}}}={\bar{\zeta }}_{f}{|}_{r_{D}=r_{\mathrm{eD}}}=0\;{\bar{\zeta }}_{F}{|}_{r_{D}=r_{\mathrm{eD}}}={\bar{\zeta }}_{f}{|}_{r_{D}=r_{\mathrm{eD}}}=0$$

The general solution to Equation (26) is as follows:

(33) $${\bar{\zeta }}_{F}=A_{1,1}I_{0}\left(\sqrt{\sigma_{1}}r_{D}\right)+B_{1,1}K_{0}\left(\sqrt{\sigma_{1}}r_{D}\right)+A_{1,2}I_{0}\left(\sqrt{\sigma_{2}}r_{D}\right)+B_{1,2}K_{0}\left(\sqrt{\sigma_{2}}r_{D}\right)\;{\bar{\zeta }}_{F}=A_{1,1}I_{0}\left(\sqrt{\sigma_{1}}r_{D}\right)+B_{1,1}K_{0}\left(\sqrt{\sigma_{1}}r_{D}\right)+A_{1,2}I_{0}\left(\sqrt{\sigma_{2}}r_{D}\right)+B_{1,2}K_{0}\left(\sqrt{\sigma_{2}}r_{D}\right)$$

The analytical solution process involves addressing the model’s non-linearity as introduced by the quadratic pressure gradient term. To achieve this, we employ a variable substitution technique, the details of which will be presented subsequently. This transformation allows us to linearize the governing equations (Equations (9) and (10)), enabling the derivation of analytical solutions in the Laplace space. The solution strategy involves separate derivations for each of the three distinct outer boundary conditions. By combining the inner boundary condition equation with each of the three outer boundary condition equations, we obtain corresponding analytical solutions in Laplace space. To transition from the Laplace domain back to the real-time domain, a critical step involves utilizing the Stehfest [37] numerical inversion algorithm. This well-established algorithm plays a pivotal role in retrieving the real-space solutions from their Laplace domain counterparts. The Stehfest algorithm employs a systematic approach to iteratively approximate the inverse Laplace transform, enabling us to convert the analytical solutions obtained in the Laplace domain into practical solutions that can be directly applied to analyze pressure behavior during well testing. Following the numerical inversion, we leverage the transformation relationship between dimensionless pressure (*p_D_*) and *ζ* established in Equation (16) to determine the real-space dimensionless pressure solution. Finally, by setting the dimensionless radial distance (*r_D_*) equal to 1, we obtain the dimensionless bottom-hole pressure solution, which represents the pressure behavior at the wellbore.

Compared with numerical simulations, the well test interpretation based on analytical models is quicker and more widely used. However, traditional analytical models of CBM wells during the injection fall-off test ignore the influence of the nonlinear problem caused by the quadratic pressure gradient, resulting in significant error in production performance analysis. In addition, the CBM reservoir is composed of natural micro-fracture and coal cleat systems, and the physical properties of the two systems are independent. However, transient pressure analysis for CBM wells during the injection fall-off test is commonly performed assuming that the CBM reservoir is only composed of the coal matrix and cleats. The influence of micro-fractures on transient pressure response is seldom considered in these models, resulting in some differences with reality. In order to solve this problem, this paper presents a novel analytical model to examine the combined effects of the quadratic pressure gradient and dual-permeability flow behavior of natural micro-fractures and coal cleats on the transient pressure response of a CBM well during the injection fall-off test. By utilizing variable substitution and Laplace transformation to linearize the mathematical model, an analytical solution is derived. Based on the solution, a series of new well test interpretation type curves are plotted, and flow regimes are observed. Moreover, the effects of coal reservoir and fracture parameters on transient pressure responses are also analyzed. The results show that the proposed model can accurately describe the nonlinear flow characteristics of CBM well during the injection fall-off test. The quadratic pressure gradient imposes effects on the intermediate flow period and the late-time pseudo-radial flow period, and the pressure and pressure derivative curves considering the quadratic pressure gradient are lower than those of the traditional linear models. Overall, the seepage of formation fluids is a nonlinear physical process, and the influence of the quadratic pressure gradient on seepage characteristics should not be overlooked. The proposed model considering the quadratic pressure gradient can accurately describe the nonlinear flow characteristics of CBM well during the injection fall-off test.

## 3. Results and Discussion

### 3.1. Flow Regime Identification

**Figure 3:**
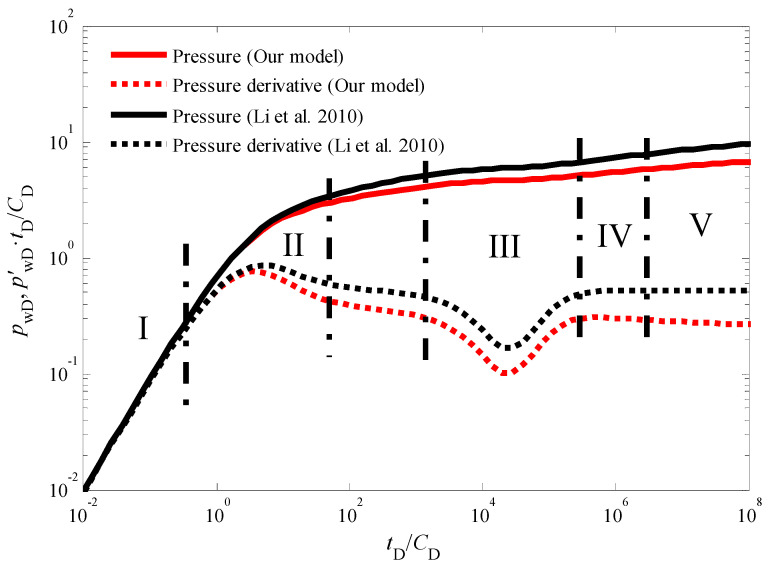
The transient pressure responses of CBM wells during the injection fall-off test in an infinite outer boundary with the quadratic pressure gradient generated by our model and ref. [26].

Figure 3 presents the standard type curves for transient pressure behavior during an injection fall-off test in a vertical CBM well. These type curves depict the dimensionless bottom-hole pressure and its derivative over dimensionless time. A close examination of Figure 3 reveals five distinct flow stages:

Stage I: Early Wellbore Storage Effect: This initial stage is characterized by the dominance of wellbore storage effects. The pressure and pressure derivative curves completely overlap, exhibiting a unit slope line on the derivative plot [38,39]. This behavior signifies that pressure changes are primarily confined to the wellbore volume during this short period.

Stage II: Skin Effect: As wellbore storage effects diminish, the influence of skin effect becomes evident. The pressure derivative curve displays a characteristic “hump” shape during this stage. The presence and severity of the hump can be correlated to the degree of formation damage or stimulation near the wellbore.

Stage III: Cleat to Fracture Flow: This stage is marked by a distinct “V” shaped trough in the pressure derivative curve. This trough signifies the transition from flow within the coal cleat system to flow within the natural fracture network. The specific characteristics of this trough can provide valuable insights into the properties of the cleat and fracture systems within the CBM reservoir.

Stage IV: Late Pseudo-Radial Flow: As the test progresses, the system reaches a state of dynamic equilibrium, characterized by late pseudo-radial flow [40]. During this stage, the pressure derivative curve deviates from the traditional “half-slope line” observed in conventional well testing analysis. This deviation is a direct consequence of the quadratic pressure gradient term incorporated into the model.

Stage V: Boundary-Dominated Flow: In the final stage, the pressure response becomes increasingly influenced by the outer boundary conditions of the reservoir [41,42,43,44]. The specific details of this stage depend on the chosen outer boundary conditions (e.g., constant pressure, no-flow) and require further analysis based on the specific reservoir geometry and production history.

**Figure 4:**
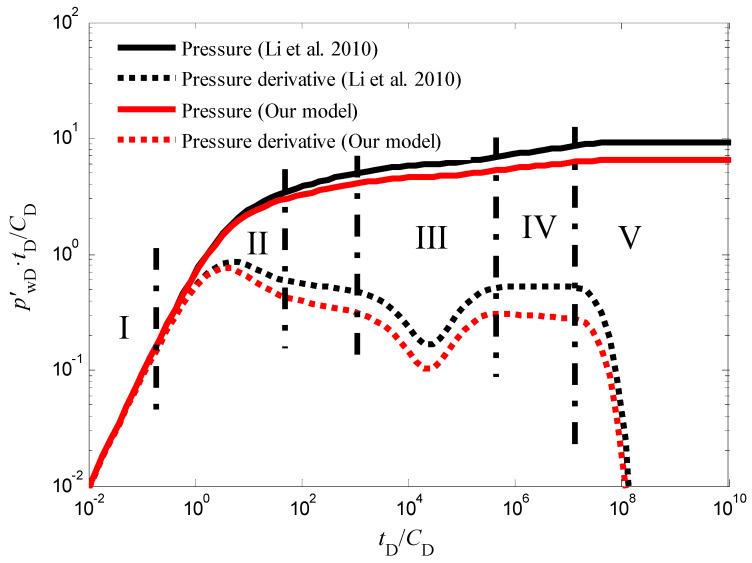
The transient pressure responses of CBM wells during the injection fall-off test in a constant pressure outer boundary with the quadratic pressure gradient generated by our model and ref. [26].

**Figure 5:**
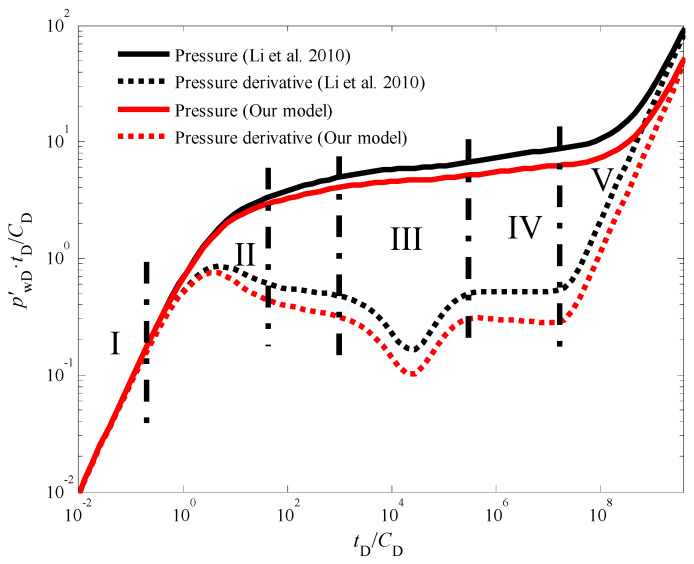
The transient pressure responses of CBM wells during the injection fall-off test in a closed outer boundary with the quadratic pressure gradient generated by our model and ref. [26].

It is important to note that the influence of the quadratic pressure gradient extends beyond the pseudo-radial flow stage. As illustrated in Figure 3 and Figure 4, the pressure derivative curve during the radial flow stage deviates from the “0.5 line rule” typically observed in conventional analysis [45,46,47]. Similarly, Figure 5 demonstrates that in the pseudo-steady-state flow stage, the pressure and pressure derivative curves, while still overlapping, no longer adhere to the “unit slope line rule” when the quadratic pressure gradient is considered. Notably, the pressure derivative is dimensionless in Figure 3 and others in this paper. The dimensionless pressure derivative is the rate of change of dimensionless pressure (i.e., the ratio of pressure to a reference pressure) over time (or dimensionless time). It is commonly used to describe the rate and pattern of pressure decline in hydrocarbon reservoirs. In addition, in well test analysis, the dimensionless pressure derivative is typically obtained by plotting the relationship between pressure (or the logarithm of pressure) and time (or the logarithm of time).

**Figure 6:**
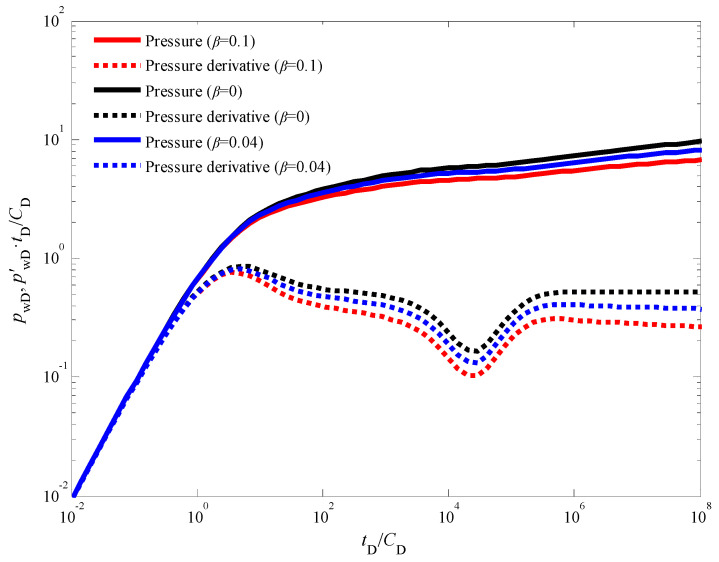
The impact of the quadratic pressure gradient on the dynamic pressure response.

Figure 6 presents double logarithmic type curves that illustrate the influence of the quadratic pressure gradient coefficient (*β*) on bottom-hole pressure behavior during an injection fall-off test in a CBM well. Curve ① represents the pressure response for a scenario with *β* = 0, which corresponds to a conventional linear seepage model. Conversely, curves ② and ③ depict the pressure response for non-zero quadratic pressure gradient coefficients *β* = 0.04 and *β* = 0.1, respectively. A key observation from Figure 6 is that the quadratic pressure gradient exerts a significant influence on the entire flow process beyond the wellbore storage effect stage (Stage I). This influence manifests as a pronounced deviation in both the bottom-hole pressure and pressure derivative curves between the non-linear (quadratic) and linear seepage models. The magnitude of the deviation is directly linked to the value of the quadratic pressure gradient coefficient. As *β* increases from 0.04 (curve ②) to 0.1 (curve ③), the impact on the pressure response becomes more pronounced, highlighting the intensifying non-linear flow characteristics. This behavior can be attributed to the fact that the quadratic pressure gradient term becomes more dominant as *β* increases. However, the pure wellbore storage effect stage (Stage I) remains unaffected by the quadratic pressure gradient because this stage primarily reflects pressure changes confined to the wellbore itself. Interestingly, the pressure and pressure derivative curves for the non-linear seepage model (considering the quadratic pressure gradient) exhibit values lower than those predicted by traditional models (assuming linear flow). This observation suggests that neglecting the quadratic pressure gradient can lead to an overestimation of both pressure and its derivative during well testing analysis. The extent of this underestimation quantified by the displacement between the curves is influenced by two factors: the dimensionless quadratic pressure gradient coefficient (*β*) and the dimensionless production time (*τ_D_*). As either *β* or *τ_D_* increases, the displacement between the curves becomes more pronounced.

### 3.2. Quantitative Specification of the Nonlinear Parameters

To quantify the discrepancies between the non-linear and linear models, we introduce two key metrics: absolute difference (DV) and relative difference (RDV). These metrics provide valuable insights into the magnitude and relative significance of the deviations observed in pressure and pressure derivative behavior between the two approaches. Table 2 and Table 3 present the calculated theoretical deviation values for β = 0.04 and β = 0.1, respectively. A consistent trend emerges from these tables: both DV and RDV exhibit a progressive increase with dimensionless production time (*τ_D_*). This increasing trend signifies that the discrepancies between the non-linear and linear models become more pronounced as the injection fall-off test progresses. Notably, RDV consistently exceeds DV, indicating that the relative difference between the models is generally larger than the absolute difference. For instance, at a dimensionless cumulative production time of *τ_D_*/*C_D_* = 103 and *β* = 0.04, the RDV of the pressure derivative curve is 16.33% greater than the RDV of the pressure curve. This observation suggests that while both pressure and pressure derivative are impacted by neglecting the quadratic pressure gradient, the relative impact on the pressure derivative is more significant. Furthermore, the deviation between the models intensifies with increasing values of the quadratic pressure gradient coefficient (*β*). This is evident from the data in Table 3, where at *τ_D_*/*C_D_* = 107, the RDV of the pressure curve for *β* = 0.1 is 28.91% larger compared to the RDV for *β* = 0.04. These observations collectively underscore the importance of considering the non-linear flow behavior caused by the quadratic pressure gradient. The proposed model, which incorporates this critical factor, provides a more accurate representation of the pressure response during injection fall-off tests in CBM wells, enabling more reliable well performance analysis and improved reservoir management strategies.

### 3.3. Sensitivity Analysis

**Figure 7:**
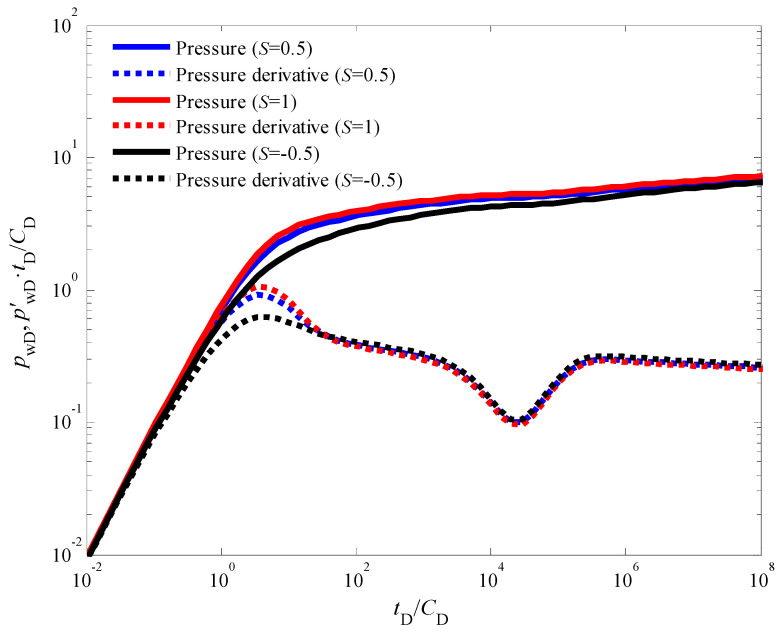
The effect of skin factor on the dynamic pressure response.

**Figure 8:**
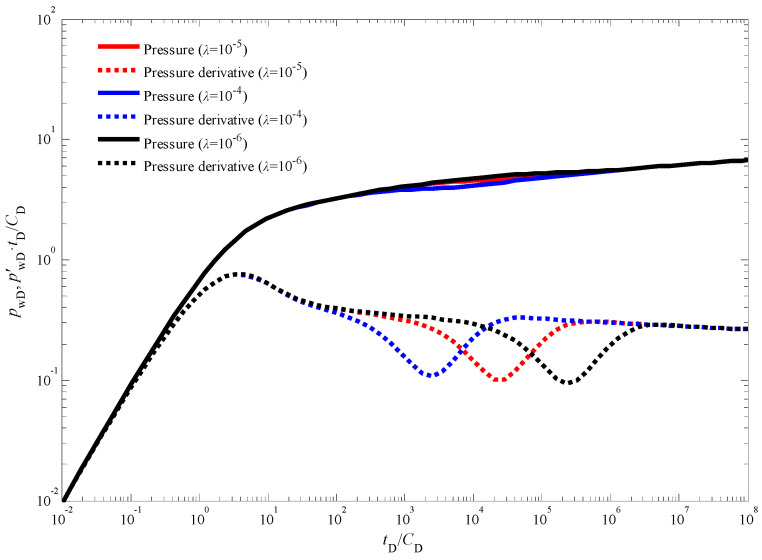
The effect of inter-porosity flow coefficient on the dynamic pressure response.

**Figure 9:**
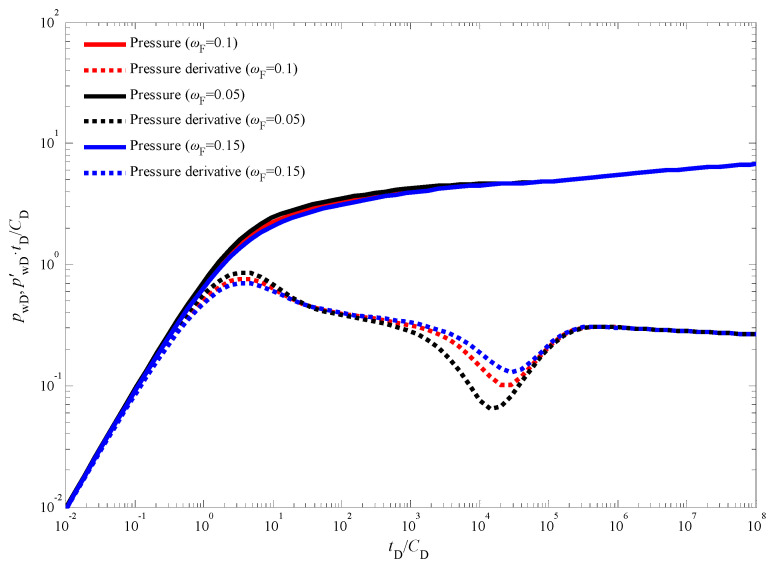
The effect of storativity ratio on the dynamic pressure response.

Figure 7 presents the influence of the skin factor on the transient pressure response during an injection fall-off test in a CBM well. The skin factor quantifies the near-wellbore formation damage or stimulation [45,46], impacting the pressure behavior in the immediate vicinity of the wellbore. A smaller skin factor corresponds to a reduced additional pressure drop near the wellbore. This translates to faster propagation of the pressure wave throughout the reservoir and a slower rate of decline in production after the injection phase. Consequently, as observed in Figure 7, wells with lower skin factors exhibit pressure and pressure derivative curves positioned at lower absolute values compared to wells with higher skin factors. It is noteworthy that despite the influence of the skin factor, the bottom-hole pressure behavior in Figure 3 still retains the characteristic features associated with a dual-porosity system. These features are manifested by the distinct “V”-shaped trough observed in the pressure derivative curve, signifying the transition from flow within the coal cleat system to flow within the natural fracture network. Figure 8 explores the impact of the inter-porosity flow coefficient on the transient pressure response. This coefficient governs the rate of fluid exchange between the coal cleat and natural fracture systems within the reservoir. A larger inter-porosity flow coefficient signifies a more rapid transfer of fluids between these two porosity systems. As illustrated in Figure 8, a higher inter-porosity flow coefficient leads to an earlier occurrence of the “V”-shaped trough in the pressure derivative curve, indicating an earlier onset of fluid crossflow from the coal cleats to the natural fractures. Finally, Figure 9 investigates the influence of the storativity ratio on the transient pressure response. The storativity ratio represents the relative amount of fluid stored within the coal cleats compared to the natural fractures at the initial moment in the reservoir [47]. A smaller storativity ratio signifies a scenario where a lesser volume of fluid is initially stored within the coal cleats and a larger volume is stored within the natural fractures. This scenario manifests as wider and deeper troughs in the pressure derivative curves, as depicted in Figure 9.

**Figure 10:**
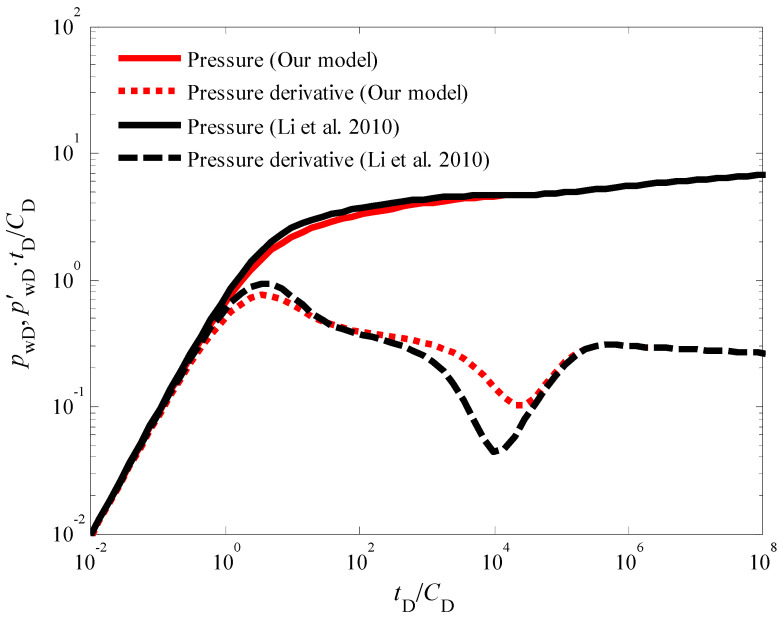
Comparison chart of characteristic curves generated by our model and ref. [26] for dual-porosity and single-porosity models in coalbed methane injection fall-off test.

Conventional coalbed methane (CBM) injection fall-off test models frequently rely on a simplified assumption: flow towards the wellbore occurs exclusively from either cleats or natural fractures within the CBM reservoir. This assumption, while computationally convenient, presents a significant departure from the complexities of actual flow dynamics observed in these formations. To bridge this gap and achieve a more realistic representation of pressure behavior, this study incorporates simultaneous flow from both cleats and natural fractures into the CBM injection fall-off test model. The analytical solution of the proposed model forms the basis for the comparison of pressure response characteristics under dual-porosity and single-porosity conditions, as illustrated in Figure 10. A close examination of Figure 10 reveals that the modified CBM injection fall-off test model, which explicitly accounts for the dual-porosity nature of the reservoir, continues to exhibit the five distinct flow stage characteristic of conventional models: early wellbore storage effect, skin effect, cleat-to-fracture flow transition, pseudo-radial flow, and boundary-dominated flow. However, under identical parameter conditions, a key distinction emerges during the cleat-to-fracture flow transition stage. The “V”-shaped trough observed in the pressure derivative curve for the dual-porosity model exhibits a shallower depth and narrower width compared to its counterpart in the conventional single-porosity model. This discrepancy can be primarily attributed to the reduced wellbore pressure drawdown experienced in the dual-porosity scenario. Due to the direct contribution of cleat flow to wellbore replenishment, the pressure decline within the dual-porosity model is less pronounced. This translates to lower pressure derivative values compared to the single-porosity model, manifesting as a shallower “V”-shaped trough in the pressure derivative curve. It is important to emphasize that during the pure wellbore storage effect stage, the reservoir remains static, with no flow occurring within the formation itself. Consequently, both cleat and fracture pressures decline in synchrony during this initial stage. This synchronicity explains why the dual-porosity and single-porosity curves completely overlap during both the wellbore storage effect and pseudo-radial flow stages.

### 3.4. Field Application

**Figure 11:**
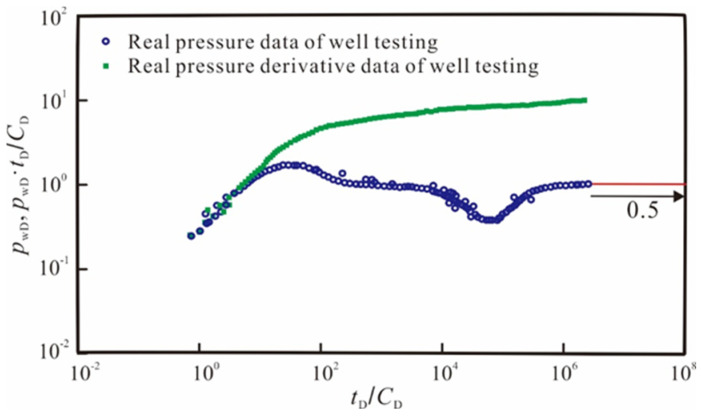
Real data of well testing.

**Figure 12:**
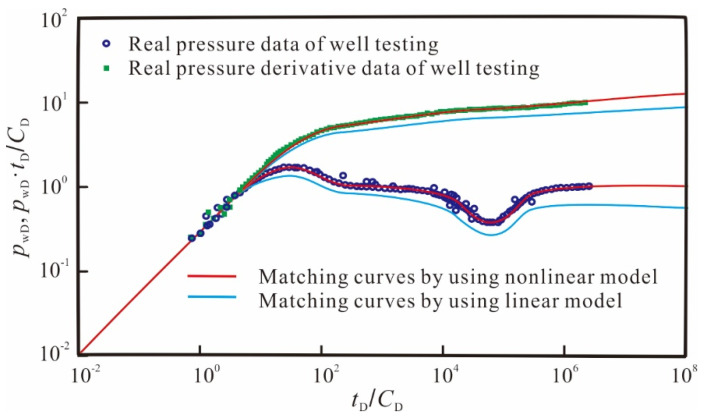
Dimensionless matching curves of well-test interpretation for the sample well.

In this section, this study utilizes the new model to conduct case analysis and application in mining fields. Firstly, we conducted dimensionless processing of the actual test data [50], as shown in Figure 11. It can be observed that the curves plotted with test data exhibit obvious nonlinear characteristics, and the pressure derivative curve during the radial flow stage deviates from the “0.5 line rule” typically observed in conventional analysis. Then, we used the conventional linear model and the theoretical curve generated by the new model to fit the measured data, as shown in Figure 12. From the results, the new model considering the quadratic pressure gradient fits the actual test data better, while the conventional linear model shows significant differences from the actual results. This observation suggests that neglecting the quadratic pressure gradient can lead to an overestimation of both pressure and its derivative during well testing analysis.

### 3.5. Model Comparison

Ultimately, it is necessary to compare the computational time/effort, accuracy of the proposed approach to a conventional linear model and also to the non-linear model without simplifications. The comparison results of the fitting between the two models and the measured data are shown in Table 4. The relative difference of the fit using the linear model reached 27.5%. However, the relative difference of the fit using the proposed model in this paper is only 5.8%.

**Figure 13:**
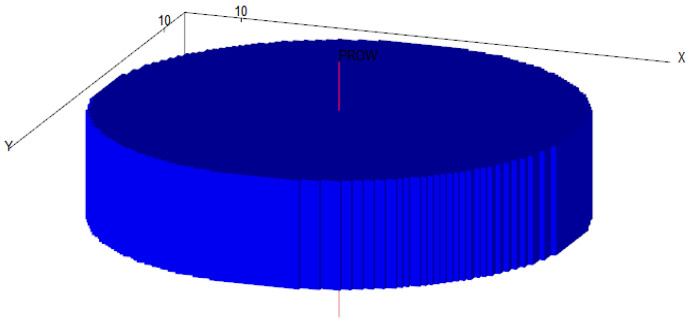
Schematic of the numerical model for an injection well in the CBM reservoir.

**Figure 14:**
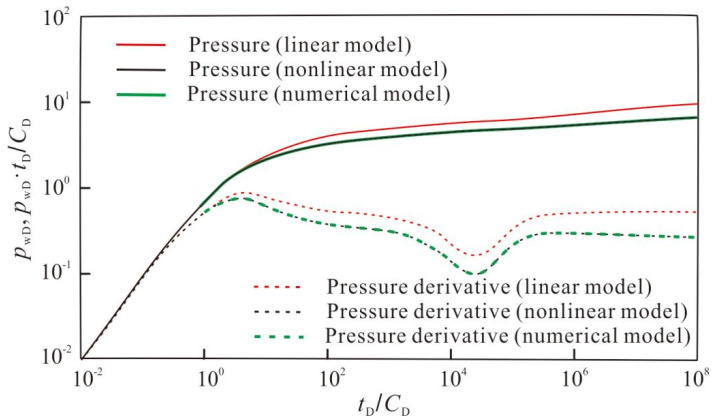
Comparison of pressure response with the linear model, the numerical model, and the proposed model in this paper.

In addition, to validate the accuracy and efficiency of the proposed model, a comparison is made with the commercial numerical simulation software tNavigator. Firstly, a dual-permeability model is established using tNavigator, as shown in Figure 13. The reservoir and fracture parameters used in the numerical model are consistent with those in the proposed model. The dimensionless pressure and pressure derivative curves obtained from the numerical model, linear model, and proposed model are compared in Figure 14. It can be found that, except for the early stage of the pressure curve, which has some deviation, the curve shapes of the numerical model and the proposed model are consistent in other flow stages. The early-stage pressure deviation is mainly caused by the radial convergence effect of cracks towards the wellbore. Numerical simulation methods usually use well models to simulate early radial flow. In this study, the model introduces the confluence skin factor to correct the bottomhole pressure and improve the accuracy of early flow simulation. Since the expression of pressure derivative has no relation to the skin, the pressure derivative curves obtained from both methods fit well throughout the entire flow stage. Currently, for numerical simulation methods, crack meshes are usually refined to obtain high-precision simulation results. After refinement, the total number of cracks meshes generally reaches tens of thousands, and the simulation time step is very small, which ultimately leads to low computational efficiency and cannot meet the high-precision requirements for early crack flow in well testing interpretation analysis. The proposed model established in this paper does not need to be divided into meshes. This greatly reduces the total number of meshes. In addition, the solution is not limited by the time step, so the early simulation accuracy is better, and the computational efficiency is higher. To obtain the calculation results shown in Figure 14, the proposed model developed in this study was used for calculation on the same platform; the time was less than 5 s, while Eclipse’s calculation time was more than 50 s. It can be seen that the proposed model studied in this paper has a significant advantage in computational efficiency.

## 4. Conclusions

This paper introduced a novel methodology for simulating the transient pressure behavior of coalbed methane (CBM) wells during injection fall-off tests. This methodology incorporates a critical term, the quadratic pressure gradient, to account for the non-linear flow characteristics observed in CBM formations. The key findings of this study are as follows:

(1) Impact of the Quadratic Pressure Gradient: The inclusion of the quadratic pressure gradient term in the model exerts a significant influence on the pressure response during the injection fall-off test. This influence is particularly pronounced during two distinct flow stages: the intermediate flow period and the late-time pseudo-radial flow period. When compared to traditional linear models that neglect the quadratic pressure gradient, the proposed model predicts lower bottom-hole pressure and pressure derivative values throughout these two stages. This observation underscores the importance of considering the non-linear flow behavior for accurate pressure response prediction in CBM wells.
(2) Wellbore Storage Effect: The wellbore storage stage, characterized by the dominance of wellbore storage effects, remains unaffected by the quadratic pressure gradient. This is because this initial stage primarily reflects pressure changes confined to the wellbore volume itself, rather than fluid flow within the formation.
(3) Influence of Dimensionless Parameters: The extent of the discrepancy between the pressure and pressure derivative curves obtained from the proposed model and those predicted by conventional linear models is governed by two key dimensionless parameters: the dimensionless quadratic pressure gradient coefficient and the dimensionless production time. As the value of the dimensionless quadratic pressure gradient coefficient increases, the deviation between the curves becomes more pronounced. This signifies a growing impact of the non-linear flow behavior on the pressure response with increasing severity of the pressure gradient. Additionally, the deviation progressively widens with increasing dimensionless production time, highlighting the growing influence of non-linear effects as the injection fall-off test progresses.
(4) Inter-Porosity and Storativity Effects: The inter-porosity flow coefficient, which governs the rate of fluid exchange between the cleat and natural fracture systems within the CBM reservoir, primarily affects the timing of the appearance of a characteristic concave-shaped trough in the pressure derivative curves. This trough signifies the transition from flow within the cleat system to flow within the fracture network. Conversely, the storativity coefficient, which represents the relative amount of fluid initially stored within the cleat and fracture systems, influences the width and depth of this concave trough in the pressure derivative curves. A smaller storativity coefficient, indicative of a lower initial fluid volume within the cleats, leads to wider and deeper troughs.

## Figures and Tables

**Table 1:** Definitions of the parameters used in this work.

Parameters	Symbol	Definition
Dimensionless pressure of natural fracture	$p_{\mathrm{FD}}$ $p_{\mathrm{FD}}$	$p_{\mathrm{FD}}=\frac{\left(k_{F}+k_{f}\right)h}{1.842\times 10^{-3}\mu_{w}qB_{w}}\left(p_{i}-p_{F}\right)$ $p_{\mathrm{FD}}=\frac{\left(k_{F}+k_{f}\right)h}{1.842\times 10^{-3}\mu_{w}qB_{w}}\left(p_{i}-p_{F}\right)$
Dimensionless pressure of cleats	$p_{\mathrm{fD}}$ $p_{\mathrm{fD}}$	$p_{\mathrm{fD}}=\frac{\left(k_{F}+k_{f}\right)h}{1.842\times 10^{-3}\mu_{w}qB_{w}}\left(p_{i}-p_{f}\right)$ $p_{\mathrm{fD}}=\frac{\left(k_{F}+k_{f}\right)h}{1.842\times 10^{-3}\mu_{w}qB_{w}}\left(p_{i}-p_{f}\right)$
Dimensionless pseudo time	$t_{D}$ $t_{D}$	$t_{D}=\frac{3.6\left(k_{F}+k_{f}\right)}{\mu_{w}{r_{w}}^{2}\left(\phi_{F}C_{\mathrm{tF}}+\phi_{f}C_{\mathrm{tf}}\right)}t$ $t_{D}=\frac{3.6\left(k_{F}+k_{f}\right)}{\mu_{w}{r_{w}}^{2}\left(\phi_{F}C_{\mathrm{tF}}+\phi_{f}C_{\mathrm{tf}}\right)}t$
Dimensionless radial distance	$r_{D}$ $r_{D}$	$r_{D}=\frac{r}{r_{w}e^{-S}}$ $r_{D}=\frac{r}{r_{w}e^{-S}}$
Dimensionless wellbore storage coefficient	$C_{D}$ $C_{D}$	$C_{D}=\frac{C_{s}}{6.2832\left(\phi_{F}C_{\mathrm{tF}}+\phi_{f}C_{\mathrm{tf}}\right)hr_{w}^{2}}$ $C_{D}=\frac{C_{s}}{6.2832\left(\phi_{F}C_{\mathrm{tF}}+\phi_{f}C_{\mathrm{tf}}\right)hr_{w}^{2}}$
Dimensionless pseudo permeability modulus	β β	$\beta =\frac{1.842\times 10^{-3}\mu_{w}qB_{w}}{\left(k_{F}+k_{f}\right)h}C_{\rho }$ $\beta =\frac{1.842\times 10^{-3}\mu_{w}qB_{w}}{\left(k_{F}+k_{f}\right)h}C_{\rho }$
The permeability ratio of the natural fracture system to the sum of fracture and cleat systems	κ κ	$\kappa =\frac{k_{F}}{k_{F}+k_{f}}$ $\kappa =\frac{k_{F}}{k_{F}+k_{f}}$
Capacitance coefficient of natural fracture	$\omega_{F}$ $\omega_{F}$	$\omega_{F}=\frac{\phi_{F}C_{\mathrm{tF}}}{\phi_{F}C_{\mathrm{tF}}+\phi_{f}C_{\mathrm{tf}}}$ $\omega_{F}=\frac{\phi_{F}C_{\mathrm{tF}}}{\phi_{F}C_{\mathrm{tF}}+\phi_{f}C_{\mathrm{tf}}}$
Capacitance coefficient of cleats	$\omega_{f}$ $\omega_{f}$	$\omega_{f}=\frac{\phi_{f}C_{\mathrm{tf}}}{\phi_{F}C_{\mathrm{tF}}+\phi_{f}C_{\mathrm{tf}}}$ $\omega_{f}=\frac{\phi_{f}C_{\mathrm{tf}}}{\phi_{F}C_{\mathrm{tF}}+\phi_{f}C_{\mathrm{tf}}}$
Inter-porosity flow factor of cleat system into natural fracture system	λ λ	$\lambda =\frac{\alpha k_{f}r_{w}^{2}}{k_{F}+k_{f}}$ $\lambda =\frac{\alpha k_{f}r_{w}^{2}}{k_{F}+k_{f}}$

**Table 2:** The theoretical deviation values between the nonlinear and linear model for *β* = 0.04.

*t*_D_/*C*_D_	*p*_wD_		*DV*	*RDV* (%)	*p’*_wD_·*t*_D_/*C*_D_		*DV*	*RDV* (%)
	Linear Models [26,48,49]	Our Model			Traditional Models	Our Model		
10	0.71	0.65	0.06	8.45	0.57	0.52	0.05	8.77
10^3^	5.14	4.57	0.57	11.09	0.49	0.41	0.08	16.33
10^7^	8.51	7.31	1.2	14.10	0.51	0.39	0.12	23.53

**Table 3:** The theoretical deviation values between the nonlinear and linear model for *β* = 0.1.

*t*_D_/*C*_D_	*p*_wD_		*DV*	*RDV* (%)	*p’*_wD_·*t*_D_/*C*_D_		*DV*	*RDV* (%)
	Linear Models [26,48,49]	Our Model			Traditional Models	Our Model		
10	0.71	0.63	0.08	11.27	0.57	0.45	0.12	21.05
10^3^	5.14	3.62	1.52	29.57	0.49	0.31	0.18	36.73
10^7^	8.51	6.05	2.46	28.91	0.51	0.28	0.23	45.10

**Table 4:** The theoretical deviation values between the nonlinear and linear model (*t*_D_/*C*_D_ = 10^2^).

*p’*_wD_·*t*_D_/*C*_D_	**Models**	**Results**	***DV***	***RDV* (%)**
	Measured data	1.547	0	0
	Linear model	1.121	0.426	27.5
	Our model	1.458	0.089	5.8

## Data Availability

Data are contained within the article.
